# Analysis of Short-term Blood Pressure Variability in Pheochromocytoma/Paraganglioma Patients

**DOI:** 10.3390/cancers11050658

**Published:** 2019-05-12

**Authors:** Valeria Bisogni, Luigi Petramala, Gaia Oliviero, Maria Bonvicini, Martina Mezzadri, Federica Olmati, Antonio Concistrè, Vincenza Saracino, Monia Celi, Gianfranco Tonnarini, Gino Iannucci, Giorgio De Toma, Antonio Ciardi, Giuseppe La Torre, Claudio Letizia

**Affiliations:** 1 Department of Translational and Precision Medicine, Unit of Secondary Arterial Hypertension, “Sapienza” University of Rome, Viale del Policlinico 155, 00165 Rome, Italy; valeria.bisogni@hotmail.it (V.B.); luigi.petramala@uniroma1.it (L.P.); gaiaoliviero2@gmail.com (G.O.); bonvimery@gmail.com (M.B.); mezzadri.1615511@studenti.uniroma1.it (M.M.); federica.kolmati@gmail.com (F.O.); antonio.concistre@gmail.com (A.C.); vincenza.saracino@gmail.com (V.S.); celi.monia@gmail.com (M.C.); gianfranco.tonnarini@uniroma1.it (G.T.); 2 Department of Internal Medicine and Medical Specialties, “Sapienza” University of Rome, Viale del Policlinico 155, 00165 Rome, Italy; gino.iannucci@uniroma1.it; 3 “Pietro Valdoni” Surgery Department, “Sapienza” University of Rome, Viale del Policlinico 155, 00165 Rome, Italy; giorgio.detoma@uniroma1.it; 4 Department of Radiological, Oncological and Anatomy-Pathological Sciences, “Sapienza” University of Rome, Viale del Policlinico 155, 00165 Rome, Italy; antonio.ciardi@uniroma1.it; 5 Department of Public Health and Infectious Diseases, “Sapienza” University of Rome, Viale del Policlinico 155, 00165 Rome, Italy; giuseppe.latorre@uniroma1.it

**Keywords:** pheochromocytoma, paraganglioma, hypertension, blood pressure variability, average real variability, weighted standard deviation

## Abstract

Data on short-term blood pressure variability (BPV), which is a well-established cardiovascular prognostic tool, in pheochromocytoma and paraganglioma (PPGL) patients is still lack and conflicting. We retrospectively evaluated 23 PPGL patients referred to our unit from 2010 to 2019 to analyze 24 h ambulatory blood pressure monitoring (24-h ABPM)-derived markers of short-term BPV, before and after surgical treatment. PPGL diagnosis was assessed according to guidelines and confirmed by histologic examination. The 24-h ABPM-derived markers of short-term BPV included: circadian pressure rhythm; standard deviation (SD) and weighted SD (wSD) of 24-h, daytime, and night-time systolic and diastolic blood pressure (BP); average real variability (ARV) of 24-h, daytime, and night-time systolic and diastolic BP. 7 males and 16 females of 53 ± 18 years old were evaluated. After surgical resection of PPGL we found a significant decrease in 24-h systolic BP ARV (8.8 ± 1.6 vs. 7.6 ± 1.3 mmHg, *p* < 0.001), in 24-h diastolic BP ARV (7.5 ± 1.6 vs. 6.9 ± 1.4 mmHg, *p* = 0.031), and in wSD of 24-h diastolic BP (9.7 ± 2.0 vs 8.8 ± 2.1 mmHg, *p* = 0.050) comparing to baseline measurements. Moreover, baseline 24-h urinary metanephrines significantly correlated with wSD of both 24-h systolic and diastolic BP. Our study highlights as PPGL patients, after proper treatment, show a significant decrease in some short-term BPV markers, which might represent a further cardiovascular risk factor.

## 1. Introduction

Pheochromocytoma (PHEO) and paraganglioma (PGL), known together as PPGL, are rare and mostly benign neuroendocrine tumors, arising from chromaffin-cells of adrenal medulla and paraganglia of the sympathetic and parasympathetic nervous system, respectively [1]. At least one-third of patients with PPGL have disease-causing germline mutations (i.e., *NF1*, *RET*, *VHL*, *SDHD*, *SDHB*, etc.) and subjects with hereditary forms typically show multifocal diseases at younger age than those with sporadic neoplasms [1,2,3,4]. The clinical presentation is so variable that PPGLs have been described as “the great masquerades” [5,6]. However, several signs and symptoms are attributed to hemodynamic and metabolic effects of the catecholamines overproduction. One of the most typical manifestations (up to 80% of cases), due to the catecholamine-excess state, is arterial hypertension, either sustained or paroxysmal [5], even though its severity does not seem to depend on the level of circulating catecholamines [6].

Although office blood pressure (BP) values remain the gold standard for the diagnosis of hypertension, the measurement of BP variability (BPV) in addition to office BP, has been demonstrated to have physiopathological and prognostic importance [7,8]. Short-term BPV refers to the BP changes that occur within a day [24 hours (24-h)], and it is influenced by several mechanisms, such as central neural factors, reflex autonomic modulation, changes in the elastic properties of arteries, humoral systems (i.e., insulin, angiotensin II, endothelin-1, bradykinin, and nitric oxide), rheological and mechanical factors [8]. Several studies have shown that higher 24-h BPV, assessed by 24-h ambulatory blood pressure monitoring (24-h ABPM), independently of mean office BP values, is clinically important, as this might increase cardiovascular events, mortality, and hypertension-mediated organ damage [9,10,11,12,13,14]. Concerning the short-term BPV in PPGL, assessed by 24-h ABPM, only outdated and small studies have been published [15,16,17,18] with contrasting results. As higher cardiovascular mortality has been reported in PPGL subjects [19], increased BPV might be a contributor for enhanced cardiovascular morbidity and mortality in this rare disease, besides the known risk factors, such as hypertension, arrhythmias, and altered glucose metabolism [20].

The aim of our study was to analyze changes in the 24-h ABPM-derived short-term BPV markers in patients affected by PPGL, before and after successful treatment by surgical removing of catecholamine-producing tumors.

## 2. Results

Table 1 shows the baseline characteristics of enrolled patients affected by PPGL. 7 males (30.4%) and 16 females (69.6%), with a median age at the diagnosis of 53 ± 18 years old, were evaluated before and after surgical tumor excision. The main signs and symptoms reported at the first visit are summarized in Table 1; the most frequent were represented by palpitation attacks, headache, sweating crises, and sustained and/or paroxysmal arterial hypertension (in 14 cases), often associated with each other. At the physical examination, we did not find cases of BP decreasing within 3 minutes of standing compared with BP from the sitting or supine position [21]. The most common comorbidities included a past history or current treatment for dyslipidemia (21.7%), coronary artery disease and/or cerebrovascular accidents (17.4%), including transient ischemic attack and ischemic/hemorrhagic stroke. One patient reported a diagnosis of diabetes mellitus type 2 and one patient history of paroxysmal atrial fibrillation.

Most of the patients had an adrenal catecholamine-producing tumor, while two women (8.6%) showed an extra-adrenal catecholamine-producing tumor in the left hypochondrium. One of these, after two years of surgical removal of the pelvic mass, revealed a recurrent form of PPGL disease with discovering a left common carotid tumor. Germline mutations were found in eight patients (34.8%) (Table 1); the most common gene involved was *VHL*, mutated in three brothers affected by von Hipple–Lindau syndrome. We also found two patients with a diagnosis of neurofibromatosis type 1 and mutation of the *NF1* gene. In two sisters we observed a *RET* gene mutation associated with a diagnosis of multiple endocrine neoplasia syndrome (MEN) type 2A. In particular, both of them presented thyroid medullary carcinoma and hyperparathyroidism, but in one patient we found an adrenal PHEO, while in the younger sister the catecholamine-producing tumor was in the left hypochondrium. In only one female, we discovered an *SDHD* gene mutation.

Surgical treatment was performed in all cases [22]. Patients with PHEO underwent laparoscopic or laparotomic adrenalectomy and patients with PGL underwent surgical excision of the extra-adrenal mass, leading to complete remission of catecholamines excess based on the normalization of biochemical and clinical features at follow-up. The histologic examination confirmed the diagnosis of PPGL; the specific features were represented by a neoplastic proliferation characterized by small nests or alveolar patterns (Zellballen), in which well-circumscribed nests of round-oval or giant or spindle-shaped nucleated neoplastic cells with eosinophil cytoplasm including catecholamine granules (“salt-and-pepper” pattern). Neoplastic cells were evaluated for chromogranin, neuron-specific enolase (NSE), and synaptophysin and CD56; the proliferation index was analyzed by Ki67 determination. All tumors had benign histological and clinical features.

In Table 2 have been reported clinical and biochemical parameters at baseline compared to follow-up from surgical treatment (mean follow-up: 26 ± 25 months). At the first visit, the levels of 24-h urinary metanephrines were 357.4 ± 190.3 μg/24-h vs. 64.1 ± 30.5 μg/24-h after surgical treatment (*p* < 0.001). Notably, the office systolic BP values were significantly lower after treatment, with a significant reduction of the number of antihypertensive drugs (2.2 ± 1.7 vs. 1.0 ± 1.0, *p* = 0.001). Moreover, we observed a modest improvement in fast glucose levels (99 ± 21 vs. 88 ± 15 mg/dl, *p* = 0.05). On the other hand, we did not find significant regression of hypertension-mediated sub-clinical vascular damage; before treatment, the mean of common carotid intima-media thickness (cIMT) at the Doppler ultrasonography was bilateral >9 mm, without plaques, and it improved only barely at the follow-up. No changes were found in the arterial stiffness variable measured by ankle-brachial index (ABI).

In Table 3, we summarized data on 24-h ABPM and 24-h ABPM-derived short-term BPV indexes. After surgical treatment, we observed a significant difference in terms of 24-h SBP values, although they were into the normal range and well-controlled after optimization of antihypertensive therapy with α-adrenergic receptor blockers. We did not find significant changes in the circadian BP rhythm, with a systolic dipping of 7.5 ± 8.0 vs. 7.7 ± 6.9 % (*p* = 0.80) and diastolic dipping of 11.7 ± 9.3 vs. 12.3 ± 7.8 % (*p* = 0.37). However, the average real variability (ARV) of both 24-h systolic and diastolic BP components, after surgical treatment, was significantly decreased (8.8 ± 1.6 vs 7.6 ± 1.3 mmHg, *p* = 0.001 and 7.5 ± 1.6 vs 6.9 ± 1.4 mmHg, *p* = 0.031, respectively), as well as the 24-h diastolic weighted SD (wSD) (Table 3). Finally, the 24-h, daytime, and night-time systolic and diastolic BP standard deviation (SD), despite not significant, showed a lowering trend (Table 3). Circadian heart rate variation remained unchanged.

A bivariate scatterplot with correlation analysis showed a direct correlation between the mean values of 24-h urinary metanephrines and (i) SD of night-time systolic BP (r = 0.45, *p* = 0.022), (ii) wSD of both 24-h systolic (r = 0.47, *p* = 0.009), and diastolic BP (r = 0.43, *p* = 0.019) performed at baseline. At multivariate analysis, the strongest predictors of 24-h systolic BP wSD were age (β = 0.566, *p* = 0.002) and baseline 24-h urinary metanephrines (β = 0.347, *p* = 0.042). For the 24-h diastolic component of wSD the strongest predictor were the 24-h urinary metanephrines values (β = 0.554, *p* = 0.006).

Lastly, we analyzed the main anthropometric and demographic data of our patients, before and after treatment, distinguishing by gender. We did not observe significant differences in terms of 24-h ABPM-derived short-term BPV indexes.

## 3. Discussion

Despite their relatively rare incidence in the general population, PPGLs are characterized by high cardiovascular morbidity and mortality [23,24]. The clinical picture associated with increased catecholamines levels, in fact, may vary from asymptomatic tumors (discovered incidentally during examinations performed due to other reasons) to life-threatening complications, such as myocardial infarction, severe heart failure, cardiomyopathy, shock, arrhythmias, and stroke. In a retrospective study, Zelinka et al. [24] observed a relatively high incidence of cardiovascular complications in PPGL subjects, reaching almost 20%. The most prevalent were arrhythmic complications (in about 10% of cases) followed by myocardial ischemia and atherosclerosis, and in about 5% of patients, cerebrovascular accidents [24]. Catecholamines excess can also lead to severe left ventricular hypertrophy. Recently, Olmati et al. presented a case of a young woman with hypertrophic cardiomyopathy secondary to a catecholamine-producing tumor and confirmed by endomyocardial biopsy, in which has been noted severe disarray of cardiomyocytes and ultrastructural evidence of contraction and necrosis of myocytes [25].

Pathogenic mechanisms that link PPGL and cardiovascular complications are multiple but mainly associated to catecholamines overproduction, which leads to increased oxygen consumption, vasoconstriction, cardiac afterload, augmented production of reactive oxygen species, cell hypertrophy through increased protein synthesis, and cardiac remodeling [23]. Arterial hypertension is a consequence of PPGL in up to 80–90% of patients [6] and it may contribute to the development and worsening of the cardiovascular profile, as well as metabolic derangements (i.e. impaired glucose tolerance), which has a reported prevalence of 25 to 75% in PHEO [26].

To evaluate cardiovascular risk in the hypertensive population, the assessment of BPV is a validated screening tool [27,28]. In physiological conditions, the short-, mid-, and long-term BPV variations have been shown to represent an adaptive mechanism to maintain homeostasis. However, sustained increases in BPV over time may also reflect alterations in cardiovascular regulatory mechanisms, which might have prognostic relevance. Clinical and population studies, using non-invasive, intermittent, reading-to-reading over 24-h monitoring provided the evidence that BPV may contribute independently to cardiovascular events prediction, over and beyond average BP [29,30,31]. Increased BPV has been associated with a higher risk of cardiovascular events, with this prediction depending on the basal risk. In low-to-moderate cardiovascular risk populations, the contribution of BPV to risk stratification has been only marginal [32], whereas in higher-risk patients, as could be those affected by PPGL, increased BPV appeared to have significant prognostic value, which might exceed that of the average BP values. Furthermore, it has been highlighted that in patients with the most common secondary forms of hypertension, such as primary aldosteronism [33], Cushing’s syndrome [34], obstructive sleep apnea [35,36], and primary hyperparathyroidism [37], the short-term BPV markers increased compared to essential hypertensive groups and decreased after specific treatments.

In our retrospective study, we evaluated data on 23 patients with a diagnosis of PHEO or PGL underwent surgery. After successfully removing the cause of excessive production of catecholamines the office systolic BP and the number of antihypertensive drugs significantly decreased. Even more important, we found changes in some markers of short-term BPV assessed by 24-h ABPM. In particular, we observed a reduction in ARV of 24-h systolic and diastolic BP. This index focuses on modifications that have been registered over short-time intervals and, thus, corrects some of the limitations of SD, which only reflects the dispersion of BP measurements around the mean [29]. The ARV has been recently demonstrated (i) to be a better estimator of 24-h BPV than other measures of dispersion, (ii) to be more useful for determining therapeutic measures aimed at controlling BPV, (iii) to have greater predictive value than the SD, after adjustment for BP and other clinical factors, for the presence and progression of subclinical organ damage [29], for total and cardiovascular mortality, and fatal combined with nonfatal cerebrovascular events [38]. Therefore, ARV of 24-h systolic and diastolic BP components is crucial to stratify the cardiovascular risk.

In our study, we also discovered a significant correlation between 24-h urinary metanephrines values and wSD of 24-h systolic and diastolic BP at baseline. Moreover, at multivariate analysis, after adjustment for sex and body mass index, the strongest predictors of 24-h systolic and diastolic wSD were the 24-h urinary metanephrines, underlying the feasible role of catecholamines overproduction in the BPV changes. The wSD represents the average of daytime and night-time BP that has been adjusted for the duration of the day and night period to account for day-night BP changes [8] and it allows to exclude the interference of night-time BP fall on overall BPV, and consents a more precise assessment of the clinical value of 24-h BPV [39]. Therefore, together with ARV, the wSD is an index unaffected by day-to-night BP changes and it should be preferred for the BPV evaluation.

According to previous research [40,41,42], we did not find changes in the systolic and diastolic BP nocturnal fall. However, various causes for the absence of dipping profile are conceivable, including sleep disturbance and psychiatric disorders, which have not been assessed by the anamnestic evaluation in our group of patients. Moreover, despite PPGLs are considered to be a curable cause of hypertension, in the long-term a substantial proportion of patients without recurrence could show higher than suggested nocturnal BP values and/or continue taking antihypertensive agents for BP control. These patients are usually (i) older, (ii) have a family history of hypertension, (iii) show higher BP values at baseline before surgical treatment, (iv) report pre-existing and associated cardiovascular risk factors (such as smoke, dyslipidemia, etc.), and (v) longer disease duration before diagnosis. In particular, referring to the last point, likewise for other forms of secondary hypertension, PPGL might lose the ability to reverse the structural vascular changes associated with secondary hypertension [43].

Lastly, we would point out that, after successful surgical treatment, the metabolic assessment significantly ameliorated with reduction of fasting glucose levels and a decreasing trend in lipid panel variables. These findings are consistent with previously reported results [44,45] and might be implicated in reducing cardiovascular risk in PPGL patients.

In summary, several studies refer to the importance of sympathetic activity and arterial baroreflexes in regulating cardiovascular variability and report other factors, including the vascular response to sympathetic stimuli, which play a role in determining the strength of BP oscillations [46,47]. Therefore, based on our results, we hypothesized that the excessive and pulsatile production of plasma catecholamines and their metabolites might have a pathogenic key role in the BPV changes. As it has been suggested by earlier researches [48,49], two types of mechanisms leading to higher BPV in PPGL patients might be involved: first, the ability of circulating catecholamines to cause rapid BP elevations; second, the high incidence of orthostatic hypotension with normally functioning baroreflex probably related to the tendency to hypovolemia [50] or because of the suppressed central sympathetic outflow [51] as a consequence of the hyperactivation of adrenergic receptors. To support these assumptions, we showed significant changes in ARV of 24-h systolic and diastolic BP and 24-h diastolic BP wSD in patients affected by PPGL after successful surgical tumor excision. These changes might represent, besides well-established and independent risk factors (i.e., hypertension, arrhythmias, and disturbances of glucose metabolism) [1,6], an additional issue for cardiovascular morbidity and mortality in this rare condition. The correlation between 24-h urinary metanephrines and systolic and diastolic wSD and the modifications mentioned above in the blood pressure variability markers at follow-up suggest the potential reversibility of cardiovascular risk by surgery in PPGL.

Thus, we strongly propose considering, in the clinical management of PPGL, as well as in normotensive PPGL subjects, the assessment of short-term 24-h ABPM-derived BPV, which, if increased, might reflect a higher risk of cardiovascular complications.

### Limitations and Strengths

Some limitations should be acknowledged. At first, the study used a retrospective design and a small sample of patients, due to the rare occurrence of catecholamine-producing tumors. Secondly, we measured only 24-h urinary metanephrines; we were able to collect plasma catecholamines and plasma metanephrines only in few cases during documented paroxysmal clinical manifestations, and, then, we could not differentiate norepinephrine- from epinephrine-secreting PPGL. Furthermore, in our population, we did not evaluate the tumor size, and we did not have any case of malignant forms; therefore, we did not understand whether the dimension of the mass and its malignant phenotype could influence BPV. Lastly, not all patients were free from other cardiovascular risk factors that could influence BPV indexes, such as smoke that was present in up to 15%, dyslipidemia (in more than 20% of cases), and previous coronary and cerebrovascular accidents.

However, hypertension in PPGLs is very complex with various clinical presentations perhaps due to the desensitization of catecholamine receptors often leading to normotension. In this context, we retain that the assessment of BPV plays a crucial role because of: (i) it allows stratifying the cardiovascular risk in this population; (ii) it could be used as a part of the screening tool of PPGL. Our findings, although only preliminary, consent to speculate a possible role of BPV in the diagnostic workup of PHEO and PGL. ARV and wSD represent better and more accurate estimators of short-term BPV than other measures of dispersion. Their record and analysis might have interesting implications in the complete evaluation and management of patients affected by PPGL, mostly in those with paroxysmal hypertension and/or in those with false-negative laboratory tests, because it may allow discovering an asymptomatic altered cardiovascular profile.

Moreover, changes in BPV markers after specific treatment may contribute to understanding better which mechanisms are implicated in the increase of cardiovascular risk in patients with catecholamine-producing tumors.

## 4. Materials and Methods

**Figure 1:**
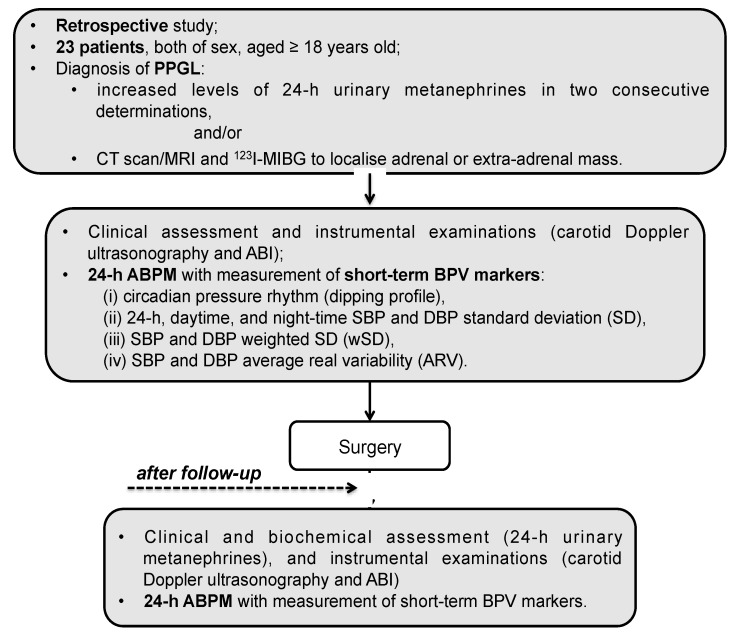
Flow-chart of the study. PPGL, pheochromocytoma and paraganglioma; CT, computed tomography; MRI, magnetic resonance imaging; MIBG, metaiodobenzylguanidine; ABI, ankle-brachial index; SBP, systolic blood pressure; DBP, diastolic blood pressure; SD, standard deviation.

From January 2011 to March 2019, we retrospectively evaluated 23 patients, both of sex and aged ≥18 years old, referred to our Secondary Hypertension Unit, University of Rome “Sapienza,” Italy (Figure 1). This study was performed according to the Declaration of Helsinki II and all participants gave informed consent.

The PPGL diagnosis was performed according to the Endocrine Society Guidelines [1], and the BPV markers were measured agreeing with the most recent recommendations [27], as described below.

All patients underwent genetic testing for the most common familial forms. At baseline and follow-up evaluations, for each patient, we collected data on anthropometric parameters and physical examination (i.e., BMI and waist circumference), past medical history, current treatment, and detailed information on cardiovascular risk (i.e., smoking, dyslipidemia, and previous cardiovascular events). To determinate hypertension-related vascular remodeling we recorded:

(i) Ankle-brachial index (ABI): we measured ABI after a 5 minutes rest in the supine position. The ABI was determined using an automated oscillometric measurement BOSO-ABI system neo (Bosch+Sohn GmbH U. Co. KG, Jungingen, Germany), which allows simultaneous arm-leg BP measurements.
(ii) Carotid Doppler ultrasonography: it determines the common carotid intima-media thickness (calculated from three separate values 1 cm proximal to the common carotid artery bifurcation in the left and right common carotid arteries), the characteristics of plaques, and the degree of stenosis [52].

### 4.1. PPGL Diagnosis

The PPGL diagnosis [1] was based on (i) clinical signs and symptoms, such as sustained or paroxysmal arterial hypertension, orthostatic hypotension, tachycardia, diaphoresis, headaches, chest pain, syncope, etc.; (ii) familial history of PPGL with specific involved genetic mutations (e.g., *VHL*, *NF1*, *RET*, *SDHD*, etc.); (iii) 24-h urinary metanephrines (measured by the RIA method; normal range: 20–345 μg/24-h), determined in two consecutive measurements; and/or (iv) abdominal magnetic resonance imaging (MRI) or computed tomography (CT) scan, subsequently confirmed by ^123^I-metaiodobenzylguanidine (^123^I-MIBG) scintigraphy, which was performed also in patients with normal values of 24-h urinary metanephrines and/or negative MRI and CT scan but high clinical suspect of PPGL.

### 4.2. Genetic Analysis

After signing of informed consent, for each patient a blood sample was obtained for the extraction of DNA according to the standardized protocol in the molecular genetics laboratory of our department [5]. DNA was extracted from the peripheral blood leukocytes of each patient with the Nucleospin blood L kit (Macherey-Nagel, Duren, Germany) and analyzed for germline mutations of *RET* (exons 10,11,13,14,15,16), *VHL* (all exons), *SDHD* (all exons), and *SDHB* (all exons). For each gene, coding regions and exon-intron boundaries, polymerase chain reaction (PCR) fragments purified with a commercial kit (PCR purification kit, Qiagen, Milan, Italy) were subject to 2% agarose gel electrophoresis with ethidium bromide staining and subsequently sequenced with a genetic analyzer (ABI PRISM 310, Applied Biosystems, Milan, Italy). For the *NF1* gene was performed a mutation analysis of the 57 exons and flanking intronic regions as well as the untranslated 50 and 30 regions of the *NF1* gene required a redesign of PCR primer pairs in order to exclude amplification of any of the 36 pseudogenes [53]. A germline intragenic mutation scanning was carried out on each of the amplicons using denaturing high-performance liquid chromatography analysis (WAVE analysis system, Transgenomics, Paris, France). Samples displaying abnormal chromatographic patterns were subjected to bidirectional direct sequencing using a MegaBACE500 DNA sequencing machine (Amersham Biosciences, Freiburg, Germany) [5].

### 4.3. Blood Pressure Variability Assessment

Short-term BPV was assessed by 24-h ABPM using Spacelabs® 90207 (SpaceLabs, Snoqualmie, WA, USA), which was carried out as a part of the procedures before diagnosis and after follow-up, in a day separate from the first office visit and after optimization of medical therapy to control BP values.

All data were acquired and analyzed according to the current recommendations [54]. Devices were calibrated periodically with a mercury sphygmomanometer and the arm cuff was positioned on the non-dominant upper limb. The between measurement intervals were 15 minutes (daytime) and 30 minutes (night-time). During each recording, subjects were required to attend at their usual daily activities, only refraining from unusual physical exercise or behavioral challenges. Only recordings rated of sufficient quality, i.e., including at least 70% of valid readings over the 24-h and at least two valid readings per hour during daytime and one valid reading per hour during night-time, were considered for the final analysis. Day and night periods were defined and corrected according to what was reported by the patient in the diary. The average daytime period was finally identified as the interval from 06:00 h to 22:00 h and the night period as the interval from 22:00 h to 06:00 h. For each recording, the mean 24-h, and day- and night-time systolic and diastolic BP have been collected. Moreover, as suggested by previous validation studies [27,55], we identified markers of short-term BPV derived by 24-h ABPM, which included:

(i) Degree of nocturnal BP fall (*dipping* pattern), calculated as [(daytime SBP − night-time SBP)/daytime SBP × 100%] for SBP and [(daytime DBP − night-time DBP)/daytime DBP × 100%] for DBP. Patients were classified as *dippers* if BP falls ≥10% and <20% of daytime average BP or *non-dippers* (fall <10%). Nocturnal BP falls ≥20% and <0% identified *“extreme” dipper* and *“reverse” dipper* subjects.
(ii) Standard deviation (SD) of 24-h, daytime, and night-time SBP and DBP;
(iii) Average of daytime and night-time SD, each weighted for the duration of the day and night periods [24-h “weighted” SD of BP (wSD)], which allows for removing the mathematical interference from night-time BP fall;
(iv) ARV for 24-h SBP and DBP, i.e., the average of the absolute differences between consecutive BP measurements over 24-h, according to a mathematical algorithm: ARV = 1^N−1^ $\sum_{k=1}^{N-1}\left({\mathrm{BP}}_{k+1}-{\mathrm{BP}}_{k}\right)$, where *N* denotes the number of valid BP measurements, and *k* is the order of measurements.

To prevent perioperative cardiovascular complications, during 2–3 weeks before surgery, patients have been treated with α-adrenergic receptor blockers (e.g., doxazosin 2 up to 16 mg daily). Preoperative co-administration of β-adrenergic receptor blockers was performed in patients with uncontrolled BP values and/or tachycardia (e.g., atenolol 50 up to 100 mg daily), only after administration of α-adrenergic receptor blockers [1]. The drugs were well tolerated and no orthostatic hypotension was observed. Moreover, all patients received preoperative volume therapy with intravenously sodium intake. The surgical intervention was performed at the Department of Surgery “Pietro Valdoni.”

### 4.4. Statistical Analysis

Data were expressed as mean and standard deviation (SD). Before statistical analysis, variables that showed a non-Gaussian distribution at Kolmogorov–Smirnov test were transformed to achieve a normal distribution and they were analyzed by non-parametric tests. Categorical variables were compared with Fisher and chi-square tests. Continuous and categorical variables were compared at baseline and follow-up by the Mann–Whitney test. ANOVA with Fisher’s Least Significant Difference post-hoc tests was used when needed. Relationships between continuous variables were assessed calculating the Pearson’s correlation coefficient. Linear regression models and multivariate analysis were performed to determine the combined effect of several variables on BPV markers. Statistical analysis was performed using SPSS software (version 24 for Mac; IBM^®^, SPSS® Statistics, Italy) and GraphPad Prism software (version 7.0, GraphPad^®^ Software Inc, San Diego, CA, USA). Significance was set at *p* < 0.05.

## 5. Conclusions

Our pilot study with its preliminary results demonstrates that (i) successful surgical treatment in patients affected by PPGL is associated with decreasing of two of the most accurate indexes of short-term blood pressure variability, (ii) there is a significant relationship between the weighted standard deviation of 24-h systolic and diastolic BP and 24-h urinary metanephrines. Therefore, it is supposable that the excessive and pulsatile production of plasma catecholamines and their metabolites in PPGLs has a role in the increased BPV markers, which is involved in the increasing of cardiovascular risk. The clinical relevance of our findings relies on the fact that these patients might take particular benefit from surgical removal of the catecholamine-secreting tumor. Moreover, short-term blood pressure variability may be used as a screening tool in the work-up of PPGLs, especially in those cases with high suspicion for which the diagnosis is challenging. Further prospective studies with a larger number of patients free from cardiovascular events and risk factors are necessary to confirm our findings.

## Figures and Tables

**Table 1:** Baseline demographic and anthropometric data. Total (*n* = 23).

**Age (Years)**	53 ± 18
**Males/Females** (%)	30.4/69.6
**Mean Follow-Up** (months)	26 ± 25
**Signs and Symptoms (%)**	
Hypertension (sustained and/or paroxysmal)	60.8
Palpitation attacks	87.5
Headache	87.5
Sweating	65.2
Syncope	13.0
Orthostatic hypotension	17.4
Chest pain	17.4
Others (i.e., abdominal pain, dizziness, etc.)	21.7
**Type of PPGL** (%)	
adrenal catecholamine-producing tumor (PHEO)	91.4
extra-adrenal catecholamine-producing tumor (PGL)	8.6
**Benign/Malignant form** (%)	100/0
**Site of Extra-Adrenal Masses** (*n*)	
Abdominal	2
Neck	1
**Multifocal forms** (*n*)	1
**Germline Mutation**	
*Total* (*n*, %)	8 (34.8)
NF1 (*n*)	2
RET (*n*)	2
VHL (*n*)	3
SDHD (*n*)	1
**Cigarettes Smoking** (%)	
yes	17.4
no	21.7
ex-smokers	13.0
n.a.	47.8
**Comorbidities** (%)	
Coronary artery disease and/or cerebrovascular accidents	17.4
Atrial fibrillation (history or current)	4.3
Diabetes mellitus	4.3
Dyslipidemia	21.7

Data expressed as mean ± standard deviation unless otherwise specified. PHEO, pheochromocytoma; PGL, paraganglioma; n.a., not available.

**Table 2:** Anthropometric, biochemical, and instrumental data at baseline compared to follow-up.

Parameters	Total (*n* = 23)		
	Baseline	Follow-Up	*p*
Age (years)	52 ± 18	54 ± 18	0.001
BMI (kg/m2)	23.0 ± 3.4	23.1 ± 3.5	0.875
Waist circumference (cm)	86.7 ± 11.3	88.4 ± 9.0	0.321
Office SBP (mmHg)	147 ± 32	131 ± 16	0.021
Office DBP (mmHg)	85 ± 20	79 ± 10	0.146
Office HR (beats/min)	75 ± 14	70 ± 14	0.754
N. of antihypertensive drugs	2.2 ± 1.7	1.0 ± 1.1	0.001
24-h urinary metanephrines (μg/24-h)	357.4 ± 190.3	64.1 ± 30.5	<0.001
Serum creatinine (mg/dL)	0.83 ± 0.25	0.98 ± 0.43	0.050
Total cholesterol (mg/dL)	217 ± 40	185 ± 27	0.099
LDL cholesterol (mg/dL)	117 ± 37	100 ± 26	0.327
HDL cholesterol (mg/dL)	79 ± 40	63 ± 22	0.034
Triglycerides (mg/dL)	115 ± 52	111 ± 39	0.861
Glycaemia (mg/dL)	99 ± 21	88 ± 15	0.050
Uric acid (mg/dL)	7.5 ± 7.7	4.9 ± 1.4	0.374
Right cIMT (mm)	0.91 ± 0.15	0.88 ± 0.22	0.551
Left cIMT (mm)	0.91 ± 0.15	0.86 ± 0.21	0.086
ABI	1.01 ± 0.19	1.07 ± 0.19	0.786

Data expressed as mean ± standard deviation unless otherwise specified. BMI, body mass index; SBP, systolic blood pressure; DBP, diastolic blood pressure; HR, heart rate; LDL, low-density lipoproteins; HDL, high-density lipoproteins; cIMT, carotid intima-media thickness; ABI, ankle-brachial index.

**Table 3:** 24-h ABPM-derived short-term blood pressure variability (BPV) markers at baseline compared to follow-up after surgical treatment.

Short-Term BPV Markers	Total (*n* = 23)		
	Baseline	Follow-Up	*p*
24-h systolic BP (mmHg)	129 ± 13	115 ± 12	0.002
24-h diastolic BP (mmHg)	73 ± 15	72 ± 9	0.329
24-h heart rate (beats/min)	75 ± 12	78 ± (9)	0.073
Systolic BP dipping (%)	7.5 ± 8.0	7.7 ± 6.9	0.808
Diastolic BP dipping (%)	11.7 ± 9.3	12.3 ± 7.8	0.837
SD of 24-h systolic BP (mmHg)	13.2 ± 4.8	12.2 ± 2.7	0.670
SD of daytime systolic BP (mmHg)	12.4 ± 3.4	11.6 ± 2.7	0.328
SD of night-time systolic BP (mmHg)	10.1 ± 4.0	9.0 ± 3.3	0.348
SD of 24-h diastolic BP (mmHg)	11.3 ± 2.7	10.2 ± 2.3	0.152
SD of daytime diastolic BP (mmHg)	10.3 ± 2.9	9.4 ± 2.5	0.057
SD of night-time diastolic BP (mmHg)	8.4 ± 2.4	7.6 ± 2.9	0.370
SD of 24-h heart rate (beats/min)	10.9 ± 3.6	11.0 ± 4.5	0.852
SD of daytime heart rate (beats/min)	10.7 ± 3.7	10.9 ± 5.0	0.988
SD of night-time heart rate (beats/min)	7.0 ± 2.6	6.7 ± 2.9	0.399
wSD of 24-h systolic BP (mmHg)	11.6 ± 3.1	10.8 ± 2.4	0.173
wSD of 24-h diastolic BP (mmHg)	9.7 ± 2.0	8.8 ± 2.1	0.050
ARV of 24-h systolic BP (mmHg)	8.8 ± 1.6	7.6 ± 1.3	0.001
ARV of 24-h diastolic BP (mmHg)	7.5 ± 1.6	6.9 ± 1.4	0.031

Data expressed as mean ± standard deviation unless otherwise specified. BP, blood pressure; SD, standard deviation; wSD, weighted standard deviation; ARV, average real variability.
